# 2506. Impact of an Innovative Community Based Contracted Pharmacy on HCV Elimination in New Jersey

**DOI:** 10.1093/ofid/ofad500.2124

**Published:** 2023-11-27

**Authors:** Corey L Rosmarin-DeStefano, Jihad Slim, Barbara Tempalski, Kevin Leyden, Emily Levaggi, Sheena Duprey, Juan Torres, Evelyn Chavez

**Affiliations:** North Jersey Community Research Inititiave (NJCRI), Denville, New Jersey; Saint Michael’s Medical Center, Newark, NJ, USA, Newark, New Jersey; NJCRI, Newark, New Jersey; NJCRI, Newark, New Jersey; NJCRI, Newark, New Jersey; NJCRI, Newark, New Jersey; NJCRI, Newark, New Jersey; NJCRI, Newark, New Jersey

## Abstract

**Background:**

North Jersey Community Research Initiative (NJCRI) in Newark, New Jersey has piloted the addition of an innovative community based contracted pharmacy to their mobile hepatitis C clinic to provide seamless care and coordination to the patient.

**Methods:**

A retrospective observational review of patients treated for hepatitis C between October 1^st^ 2020 and Sept 31^st^ 2022 at North Jersey Community Research Initiative (NJCRI), dividing patients in group 1: patients required by their insurance to fill up prescriptions through Managed Medicaid’s Preferred Specialty Pharmacies (PSP), which are corporate mail-order pharmacies and group 2: those who could fill their prescriptions with a local contracted community based pharmacy. We had two primary endpoints comparing those 2 groups: 1- Difference in wait time from the provider’s visit to starting therapy; 2- Difference in percentage of those completing therapy.

**Results:**

NJCRI prescribed hepatitis C treatment to 614 patients during the study period, group 1 had 138 patients (22.4%) and group 2 had 476 patients (78%). The average time to start of therapy was 36 days for group 1 compared to 15 days for group 2. Group 1 on average have a longer wait time to start treatment therapy. Group 2 were more likely to complete therapy utilizing local pharmacies contracted by NJCRI. Patients who started and completion treatment was 81% in-group 1 and 92% for group 2 (*p*=0.0003). Percentage LTFU after starting therapy was 19% for group 1 and 8% for group 2.

**Conclusion:**

Our study shows that community based pharmacy settings positively affect patient care and adherence because of the convenience and support to the patients as well as the providers. Restrictions of filling medications through corporate mail-order pharmacies.

**Disclosures:**

**Jihad Slim, MD, FACP**, ViiV Healthcare: Advisor/Consultant|ViiV Healthcare: Grant/Research Support

